# Patient factors associated with telehealth quality and experience among adults with chronic conditions

**DOI:** 10.1093/jamiaopen/ooae026

**Published:** 2024-03-19

**Authors:** Esther Yoon, Scott Hur, Laura M Curtis, Julia Yoshino Benavente, Michael S Wolf, Marina Serper

**Affiliations:** Division of General Internal Medicine & Geriatrics, Center for Applied Health Research on Aging, Feinberg School of Medicine, Northwestern University, Chicago, IL 60611, United States; Division of General Internal Medicine & Geriatrics, Center for Applied Health Research on Aging, Feinberg School of Medicine, Northwestern University, Chicago, IL 60611, United States; Division of General Internal Medicine & Geriatrics, Center for Applied Health Research on Aging, Feinberg School of Medicine, Northwestern University, Chicago, IL 60611, United States; Division of General Internal Medicine & Geriatrics, Center for Applied Health Research on Aging, Feinberg School of Medicine, Northwestern University, Chicago, IL 60611, United States; Division of General Internal Medicine & Geriatrics, Center for Applied Health Research on Aging, Feinberg School of Medicine, Northwestern University, Chicago, IL 60611, United States; Division of Gastroenterology and Hepatology, University of Pennsylvania Perelman School of Medicine, Philadelphia, PA, United States

**Keywords:** telemedicine, healthcare disparities, quality of health care

## Abstract

**Objective:**

To evaluate patient-reported experiences of telehealth and disparities in access, use, and satisfaction with telehealth during the COVID-19 pandemic.

**Materials and methods:**

We examined data from the fifth wave of the COVID-19 & Chronic Conditions (C3) study conducted between December 2020 and March 2021.

**Results:**

Of the 718 participants, 342 (47.6%) reported having a telehealth visit within the past 4 months. Participants who had a recent telehealth visit were younger, reported worse overall health and chronic illness burden, and living below poverty level. Among participants who had a telehealth visit, 66.7% reported telephone visits and most participants (57.6%) rated telehealth quality as better-or-equal-to in-person visits. Inadequate health literacy was associated with lower likelihood of reporting telehealth quality and usefulness. In multivariable analyses, lower patient activation (adjusted odds ratio (AOR) 0.19, 95% CI, 0.05-0.59) and limited English proficiency (AOR 0.12, 95% CI, 0.03-0.47) were less likely to report telehealth as being better than in-person visits; lower patient activation (AOR 0.06, 95% CI, 0.003-0.41) and income below poverty level (AOR 0.36, 95% CI, 0.13-0.98) were associated with difficulty remembering telehealth visit information.

**Discussion:**

Most participants reported usefulness and ease of navigating telehealth. Lower socioeconomic status, limited English proficiency, inadequate health literacy, lower educational attainment, and low patient activation are risks for poorer quality telehealth.

**Conclusion:**

The COVID pandemic has accelerated the adoption of telehealth, however, disparities in access and self-reported visit quality persist. Since telemedicine is here to stay, we identify vulnerable populations and discuss potential solutions to reduce healthcare disparities in telehealth use.

## Background and significance

The COVID-19 pandemic accelerated the pace of telehealth adoption in the United States through regulatory waivers and loosening of restrictions on interstate licensure or site of telehealth delivery.^1^ A growing body of research has investigated how telemedicine affects patient care and ways to better optimize these services. However, studies have focused on telehealth access and individual-level factors associated with perceived telehealth quality remain understudied.^2–7^

Despite the potential clinical benefits of telemedicine, there are several barriers that may limit utility. Disparities in access and usability of technology-based interventions, smartphones, patient portals, and internet/broadband have been well documented among racial and ethnic minorities, patients with limited English proficiency (LEP), older adults, and rural communities.^8–11^ Differences in use of patient portals have also been documented prior to the pandemic, with men, lower educational attainment, LEP, preference for in-person communication, and not having a primary care clinician being more likely among portal nonusers.^12^^,^^13^ Older adults can face additional challenges with telehealth, and though generally have positive attitudes towards emerging technologies, may demonstrate a lack of familiarity or confidence in internet and health information technology limiting telehealth utility.^14–16^ With the expiration of the public health emergency in May 2023, long-term telehealth reimbursement remains uncertain while little data exist regarding its clinical effectiveness, optimal clinical use, and the patient experience. Studies so far have indicated overall high patient satisfaction and acceptability with telehealth visits, including convenience and provider communication; however, experiences differed across sociodemographic characteristics (eg, gender, age, and socioeconomic status) and modality of telehealth.^17–20^ Patient-reported experience measures (PREMs) have typically focused on overall satisfaction of care, with usefulness, ease-of-use, and reliability as primary measurements, with limited considerations of perceived effectiveness of visit quality.

To date, limited research links patient experiences of telehealth to sociodemographic and psychosocial characteristics, yet to optimize telehealth visit quality, understanding potential barriers is important for clinicians and policymakers to optimize telehealth use. To fill these gaps, we examined: sociodemographic factors associated with PREMs in a diverse sample of middle-aged and older adults with at least one chronic condition who received care at academic practices and federally qualified health centers (FQHCs) in Chicago, IL.

## Methods

The COVID-19 & Chronic Conditions (C3; R01AG030611-S1) study is an ongoing, longitudinal, telephone-based survey of participants who are currently active in 1 of 5 ongoing, primary care based, National Institutes of Health (NIH)-funded studies (R01AG030611; R01AG046352; R01DK110172; R01HL126508; R01NR015444; See Appendix Table SA). The survey objective was to track the experiences of middle-aged and older adults, with underlying health conditions that placed them at higher risk for infection and adverse outcomes from COVID-19, through the course of the pandemic. The parent studies were chosen due to enrollment of participants that would have greater risk for COVID-19 (eg, largely middle-aged or older adult participants, with 1 or more chronic conditions).

### Procedure

Participants in parent studies were recruited from multiple academic and community health center performance sites in Chicago, IL: (1) Northwestern Memorial Healthcare (NMHC), consisting of multiple, large academic practices; (2) Access Community Health Network (ACCESS), a Public Health Service 330-funded network of FQHCs; and (3) Erie Family Health Center, a network of FQHCs comprised 12 large health centers affiliated with the AllianceChicago Electronic Health Record (EHR) user community. The expanded C3 cohort for timepoint 3, 5, and 7 (ie, T3, T5, and T7 respectively) included participants from (4) Mount Sinai Icahn School of Medicine academic practice in New York, New York. All sites serve diverse patient populations and have centralized EHR systems (Epic and GE Centricity [AllianceChicago only]).

Eligible participants were identified via EHR queries, sending letters to eligible participants describing the study, telephoning eligible patients who did not opt out of being contacted, screening patients for eligibility, scheduling baseline interviews. Common exclusion criteria for all studies were having severe or uncorrectable cognitive, visual, or hearing impairment. The C3 study targeted study participants who had an interview completed between 2018 and March 2020, which helped to ensure the data collected previously in the parent study was most current. Detailed information on the C3 study procedures has been previously published.^21^ Northwestern University’s Institutional Review Board serves as the single IRB of record, and all research staff maintain Human Subjects Training (Collaborative Institutional Training Initiative, ie, CITI).

### Data collection

**Figure 1.**
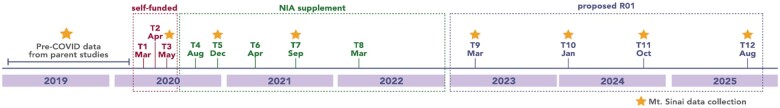
C3 study timepoints.

The C3 cohort was recruited from a subset of patients enrolled in the parent studies, which had uniform data collected on a range of patient-reported outcomes within 1 year prior to COVID-19, as well as EHRs and pharmacy records. This study used data from T5 (December 2020) time point (Figure 1).

### Measurement

The C3 study collected self-reported information on patient psychosocial characteristics, COVID-19 related beliefs and actions, health and lifestyle behaviors, health services use, and mental and physical health (Appendix Table SB).

#### Sociodemographic and psychosocial characteristics

All parent studies had uniform collection of patient information, including demographics (age, sex, race, ethnicity), socioeconomic status (SES) (household income, number in household, educational attainment, employment status, and health insurance), self-reported chronic conditions, and a 1-item, self-reported overall health (excellent, very good, good, fair, or poor). Measures of SES are routinely collected during C3 waves to note any changes to employment or income. In addition, the C3 survey included measures of other factors, including:

##### Social support

Participants’ perceived adequacy of tangible social support was evaluated using a 2-item social support scale as part of the parent studies and the C3 battery.^22^

##### Self-efficacy

The Self-Efficacy for Managing Chronic Disease 6-item Scale covers multiple domains of chronic disease self-management, including symptom control, role function, emotional functioning and communicating with physicians, and inquires about patient confidence in completing each activity.^23^

##### Health literacy

All studies also included health literacy, measured by the Newest Vital Sign (NVS). The NVS is a reliable screening tool used to determine risk for limited health literacy by asking questions about a nutrition label. Patients are given a copy of a nutrition label and asked 6 questions about how they would interpret and act on the information contained on the label. The number of correct responses is summed to produce a health literacy score ranging from 0 to 6. Scores are classified in terms of likelihood of limited literacy (0-1: likely limited; 2-3: possibly limited; 4-6: adequate).^24^

##### Patient activation

The Consumer Health Activation Index (CHAI) was used to assess patients’ degree of “activation” or motivation to participate in healthcare decisions and actions.^25^

#### Telehealth PREMs

The C3 telephone survey at T5 used an array of self-report questions evaluating participants’ telehealth experiences, including satisfaction and preferences for future clinical visits, developed by Polinski et al.^26^ These survey questions, response choices, and operationalization of PREMs are provided in Appendix Table SC. To evaluate self-reported telehealth use, we first asked C3 participants if they had telemedicine appointments in the past 4 months and how many telemedicine appointments they had, as well as whether the visits were via video or telephone. Additional outcome measures included self-reported difficulty and satisfaction with telehealth services. Participants were asked how telehealth visits compared to in-person visits, to comment on difficulty remembering visit instructions, and perceived usefulness of telehealth once the COVID-19 pandemic is over.

### Analysis plan

Statistical analysis was conducted using RStudio version 3.6.1. Appropriate descriptive statistics (eg, percentage, frequency, median) were performed on all patient variables. Univariable analysis was conducted to determine if there were any statistically significant demographic disparities between the following telehealth experience outcomes: (1) participants who had versus did not have a telehealth visit in the past 4 months, (2) who reported higher versus lower satisfaction of telehealth, and (3) who reported higher versus lower difficulty of remembering information discussed during telehealth services, (4) higher versus lower perceived usefulness of telehealth visits once COVID is over.

For categorical variables, data were analyzed using chi-square tests, or Fishers-exact test when sample size was low. Telehealth experience outcomes with Likert scale responses were collapsed into binary measures. Quality of telehealth compared to in-person visits (better, just as good, worse, not sure) was dichotomized as better/just as good and worse/not sure. Difficulty of remembering information discussed during telehealth services (very easy, somewhat easy, somewhat difficult, very difficult) was dichotomized as very/somewhat easy and very/somewhat difficult. Perceived usefulness of telehealth visits once COVID is over (very useful, somewhat useful, neutral, not useful) was dichotomized as very/somewhat useful and neutral/not useful. Data were dichotomized for ease of interpretation. As all outcomes were dichotomous, logistic regression models were performed for telehealth experience outcomes. All models were adjusted for any covariates associated with the outcomes in univariable analysis at *P*<.05, and unadjusted and adjusted odds ratios (ORs and AORs, respectively) were reported.

## Results

Of the 718 participants enrolled in the C3 study at T5, 342 (47.6%) reported having a telehealth visit within the past 4 months. Patient characteristics of this subsample are presented in Table 1.

**Table 1.** Characteristics of participants who had versus did not have a telehealth visit in past 4 months.

	All participants (*N* = 718)		Those who had telehealth visit in past 4 months (*n* = 342)		Those who have NOT had telehealth visit in past 4 months (*n* = 372)		
Participant characteristics	Count	%	Count	%	Count	%	*P*-value
Age (mean, SD)	65.8 (11.1)		64.6 (11.9)		66.8 (10.2)		.03^a^
Age group							.02^a^
<60 years	188	26.2	105	55.9	81	43.1	
60-69 years	244	34.0	115	47.1	126	51.6	
≥70 years	289	40.3	122	42.2	165	57.1	
CHAI (patient activation)							.42
High activation	52	7.2	28	53.8	24	46.2	
Moderate activation	283	39.4	138	48.8	145	51.2	
Low activation	351	48.9	158	45.0	189	53.8	
Gender							.89
Male	251	35.0	122	48.6	128	51.0	
Female	467	65.0	220	47.1	244	52.2	
NVS (health literacy)							.12
Adequate (NVS≥4)	271	37.7	128	47.2	143	52.8	
Inadequate (NVS≤3)	243	33.8	130	53.5	111	45.7	
Primary care setting							.34
Academic	538	74.9	249	46.3	286	53.2	
Federally qualified health center	180	25.1	93	51.7	86	47.8	
Health insurance							<.01^a^
Private	130	18.1	53	40.8	76	58.5	
Medicare or Medicare+private supplement	251	35.0	130	51.8	121	48.2	
Medicaid or Medicaid+private supplement	163	22.7	91	55.8	71	43.6	
Self-reported overall health							<.01^a^
Excellent	77	10.7	24	31.2	51	66.2	
Very good	220	30.6	95	43.2	125	56.8	
Good	268	37.3	141	52.6	126	47.0	
Fair/poor	153	21.3	82	53.6	70	45.8	
Number of chronic conditions							<.01^a^
≥3	403	56.1	215	53.3	187	46.4	
<3	315	43.9	127	40.3	185	58.7	
Hispanic							.87
No	434	60.4	217	50.0	215	49.5	
Yes	110	15.3	57	51.8	53	48.2	
Race							.36
Black/African American	334	46.5	83	24.9	80	24.0	
White/Caucasian	165	23.0	176	106.7	158	95.8	
Other	29	4.0	12	41.4	17	58.6	
Highschool graduate							.93
No	156	21.7	73	46.8	82	52.6	
Yes	562	78.3	269	47.9	290	51.6	
Education level							.13
HS or less	156	21.7	73	46.8	82	52.6	
Some college or technical	172	24.0	95	55.2	76	44.2	
College graduate	390	54.3	174	44.6	214	54.9	
Limited English proficiency							.76
No	657	91.5	311	47.3	342	52.1	
Yes	61	8.5	31	50.8	30	49.2	
Marital status							.94
Currently married	276	38.4	136	49.3	138	50.0	
Not currently married	372	51.8	181	48.7	190	51.1	
Employment status							.64
Not currently working	542	75.5	261	48.2	278	51.3	
Currently working	175	24.4	80	45.7	94	53.7	
Below poverty level							.01^a^
No	543	75.6	244	44.9	297	54.7	
Yes	169	23.5	96	56.8	71	42.0	
Access to video-enabled device							
Phone	559	77.9	249	44.5	278	49.7	.05^a^
Laptop/computer	472	65.7	279	59.1	257	54.4	.16
Tablet	312	43.5	213	68.3	159	51.0	.67
None	72	10.0	29	40.3	42	58.3	.11
Access to internet							.22
No	55	7.7	24	43.6	30	54.5	
Yes	661	92.1	318	48.1	341	51.6	

a Indicates statistically significant differences between groups (*P*-value≤.05).

The median age was 65.8 years (range 23-91), 35.0% (251/718) were male, and 46.5% (334/718) were African American. There were no significant differences between participants who had or did not have a telehealth visit within the past 4 months by gender, race, education, health literacy, primary care setting, English proficiency, marital status, or employment status. Participants who did not report a recent telehealth visit were older (mean 66.8 (SD 11.1) vs 64.6 (SD 11.9) years old; *P* = .03), more likely to have private insurance (58.5% vs 40.8%; *P* < .01), more likely to report better overall health (66.2% vs 31.2%; *P* < .01), more likely to have a lower chronic illness burden (46.4% had ≥3 chronic conditions vs 53.3%; *P* < .01), and more likely to be living above poverty level (54.7% vs 44.9%; *P* < .01).

Among participants with a recent telehealth visit, the average number of visits was 2.61 (range of 1-22), and 66.7% (228/342) reported that their most recent visit was via telephone. More than half (57.6%, 197/342) reported that the quality of telehealth was better or just as good as in-person visits. Most participants also reported ease of navigating telehealth visits: 88.9% (304/342) reported it was very or somewhat easy to describe their current health during a telehealth appointment, and 84.8% (290/342) reported it was very or somewhat easy to remember information discussed during their telehealth appointment. Most of the participants perceived telehealth as useful during the pandemic (80.4%, 275/342) and once the pandemic is over (75.1%, 257/342).

### Characteristics of recent telehealth use (video vs telephone)

A total of 66.6% (228/342) of participants reported their most recent telehealth visit being via telephone. As shown in Table 2, individuals were less likely to report having a recent video versus telephone telehealth visit if they had inadequate health literacy (OR 0.39, 95% CI, 0.23-0.66), received primary care at a FQHC (OR 0.29, 95% CI, 0.16-0.54), less than a high school education (OR 0.49, 95% CI, 0.27-0.90), LEP (OR 0.36, 95% CI, 0.13-0.95), were not married (OR 0.60, 95% CI, 0.37-0.96), and reported income below poverty level (OR 0.49, 95% CI, 0.29-0.84).

**Table 2.** Likelihood of most recent telehealth visit being video.

Those who had telehealth visit in past 4 months (*n* = 342)	Likelihood of most recent telehealth visit being video			
Participant characteristics	Telephone (*n*)	Video (*n*)	Unadjusted OR (95% CI)	*P*-value
Age group				.07
<60 years	61	44		
60-69 years	83	32	0.53 (0.30-0.94)	
≥70 years	84	38	0.63 (0.36-1.08)	
CHAI (patient activation)				.77
High activation	17	11		
Moderate activation	92	46	0.77 (0.33-1.78)	
Low activation	107	51	0.74 (0.32-1.69)	
Gender				.11
Male	74	48		
Female	154	66	0.66 (0.42-1.05)	
NVS (health literacy)				<.01^a^
Adequate (NVS≥4)	71	57		
Inadequate (NVS≤3)	99	31	0.39 (0.23-0.66)	
Primary care setting				<.01^a^
Academic	150	99		
Federally qualified health center	78	15	0.29 (0.16-0.54)	
Health insurance				.08
Private	28	25		
Medicare or Medicare+private supplement	88	42	0.53 (0.28-1.03)	
Medicaid or Medicaid+private supplement	64	27	0.47 (0.23-0.95)	
Self-reported overall health				.23
Excellent	13	11		
Very good	59	36	0.72 (0.29-1.78)	
Good	96	45	0.55 (0.23-1.33)	
Fair/poor	60	22	0.43 (0.17-1.11)	
Number of chronic conditions				.84
≥3	152	72		
<3	86	41	0.93 (0.58-1.48)	
Hispanic				.11
No	137	89		
Yes	43	14	0.56 (0.29-1.08)	
Race				.77
White/Caucasian	113	63		
Black/African American	55	28	0.91 (0.53-1.58)	
Other	9	3	0.60 (0.16-2.29)	
Highschool graduate				.03^a^
Yes	171	98		
No	57	16	0.49 (0.27-0.90)	
Limited English proficiency				.05^a^
No	202	109		
Yes	26	5	0.36 (0.13-0.95)	
Marital status				.04^a^
Currently married	82	54		
Not currently married	130	51	0.60 (0.37-0.96)	
Employment status				.07
Currently working	46	34		
Not currently working	181	80	0.60 (0.36-1.00)	
Below poverty level				.01^a^
No	152	92		
Yes	74	22	0.49 (0.29-0.84)	
Access to video-enabled device				.01^a^
Yes	199	114		
No	29	0	0.03 (0.002-0.49)	
Access to internet				.04^a^
Yes	207	111		
No	21	3	0.27 (0.08-0.91)	

a Indicates statistically significant differences between groups (*P*-value≤.05).

Individuals were less likely to have a video telehealth visit if they did not have access to the internet at home (OR 0.27, 95% CI, 0.08-0.91), such as Wi-Fi or data plan through a phone, or access to any video-enabled device (OR 0.03, 95% CI, 0.002-0.49), this included their own or a family member’s device.

### Telehealth PREMs

Factors associated with telehealth PREMs in univariable analyses and multivariable analyses are shown in Table 3.

**Table 3.** Bivariate and multivariable analyses of factors associated with telehealth PREMs.

	Likelihood of reporting telemed being better or just as good as in-person visits			Likelihood of reporting very or somewhat easy to remember information discussed during telehealth visit			Likelihood of reporting usefulness of telehealth visits once COVID is over		
Participant characteristics	OR (95% CI)	AOR (95% CI)	*P*-value	OR (95% CI)	AOR (95% CI)	*P*-value	OR (95% CI)	AOR (95% CI)	*P*-value
Age group									
<60 years									
60-69 years	0.93 (0.55-1.60)	9.08 (0.61-152.34)	.50	1.03 (0.48-2.21)	1.31 (0.47-3.74)	.61	0.93 (0.50-1.71)	1.19 (0.54-2.63)	.67
≥70 years	0.90 (0.53-1.53)	0.77 (0.36-1.63)	.28	0.80 (0.39-1.65)	0.62 (0.19-2.00)	.43	0.92 (0.50-1.68)	2.01 (0.72-5.90)	.19
CHAI (patient activation)									
High activation	reference	reference	reference	reference	reference	reference	reference	reference	reference
Moderate activation	0.57 (0.23-1.38)	**0.24 (0.07-0.75)**	**.02** ^a^	0.30 (0.04-2.40)	0.17 (0.01-1.19)	.13	1.20 (0.47-3.09)	0.83 (0.23-2.61)	.77
Low activation	0.45 (0.19-1.09)	**0.19 (0.05-0.59)**	**.01** ^a^	0.14 (0.02-1.07)	**0.06 (0.003-0.41)**	**.02** ^a^	0.89 (0.35-2.25)	0.59 (0.16-1.86)	.39
Gender									
Male	reference	reference	reference	reference	reference	reference	reference	reference	reference
Female	**0.62 (0.39**-**0.97)**^a^	**3.19 (1.67-6.27)**	**<.01** ^a^	0.62 (0.32-1.20)	0.54 (0.19-1.37)	.21	0.79 (0.47-1.34)	1.09 (0.52-2.24)	.82
NVS (health literacy)									
Adequate (NVS≥4)	reference	reference	reference	reference	reference	reference	reference	reference	reference
Inadequate (NVS≤3)	**0.60 (0.36**-**0.98)**^a^	0.85 (0.41-1.73)	.65	**0.45 (0.22-0.90)** ^a^	0.47 (0.16-1.32)	.16	**0.47 (0.26-0.84)** ^a^	0.48 (0.21-1.08)	.08
Primary care setting									
Academic	reference	reference	reference	reference	reference	reference	reference	reference	reference
Federally qualified health center	**0.56 (0.35**-**0.91)**^a^	1.13 (0.47-2.73)	.79	**0.44 (0.24-0.82)** ^a^	0.58 (0.17-2.01)	.39	0.64 (0.38-1.09)	1.13 (0.44-2.96)	.80
Health insurance									
Private	reference	reference	reference	reference	reference	reference	reference	reference	reference
Medicare or Medicare+private supplement	0.80 (0.41-1.55)	0.96 (0.40-2.27)	.93	0.54 (0.19-1.52)	1.26 (0.32-4.55)	.73	0.54 (0.23-1.28)	0.62 (0.21-1.69)	.36
Medicaid or Medicaid+private supplement	0.50 (0.25-1.01)	0.72 (0.25-3.75)	.52	0.42 (0.15-1.21)	2.66 (0.59-11.85)	.20	0.42 (0.18-1.01)	0.64 (0.19-2.02)	.46
Self-reported overall health									
Excellent	reference	reference	reference	reference	reference	reference	reference	reference	reference
Very good	1.07 (0.43-2.66)	1.01 (0.26-3.75)	.99	0.99 (0.26-3.82)	1.42 (0.15-8.56)	.72	0.75 (0.23-2.44)	0.83 (0.11-3.92)	.83
Good	0.96 (0.40-2.32)	1.22 (0.33-4.32)	.76	1.12 (0.30-4.16)	2.04 (0.22-12.06)	.47	0.61 (0.19-1.89)	0.60 (0.08-2.63)	.54
Fair/poor	0.87 (0.35-2.18)	1.48 (0.36-5.89)	.58	0.41 (0.11-1.53)	1.21 (0.13-7.79)	.85	0.43 (0.13-1.39)	0.56 (0.07-2.68)	.50
Number of chronic conditions									
<3	reference	reference	reference	reference	reference	reference	reference	reference	reference
≥3	0.77 (0.49-1.20)	0.78 (0.38-1.54)	.48	0.64 (0.35-1.17)	1.35 (0.51-3.48)	.54	0.75 (0.45-1.23)	**2.11 (0.99-4.62)**	**.05** ^a^
Hispanic									
No	reference	reference	reference	reference	reference	reference	reference	reference	reference
Yes	**0.37 (0.20-0.67)** ^a^	0.67 (0.26-1.71)	.39	**0.40 (0.20-0.79)** ^a^	0.36 (0.10-1.29)	.11	0.97 (0.49-1.91)	1.90 (0.65-6.54)	.27
Race									
Black/African American	reference	reference	reference	reference	reference	reference	reference	reference	reference
White/Caucasian	0.78 (0.46-1.34)	1.18 (0.57-2.45)	.65	0.60 (0.28-1.27)	0.48 (0.15-1.43)	.20	1.35 (0.74-2.49)	1.23 (0.54-2.79)	.62
Other	0.43 (0.12-1.46)	0.70 (0.15-3.19)	.64	1.51 (0.18-12.94)	1.14 (0.12-28.22)	.92	**0.26 (0.07-0.90)** ^a^	0.24 (0.05-1.16)	.07
Highschool graduate									
No	reference	reference	reference	reference	reference	reference	reference	reference	reference
Yes	0.70 (0.42-1.18)	1.21 (0.59-2.46)	.60	0.55 (0.29-1.06)	1.35 (0.53-3.38)	.52	0.77 (0.43-1.38)	1.09 (0.51-2.26)	.83
Limited English proficiency									
No	reference	reference	reference	reference	reference	reference	reference	reference	reference
Yes	**0.15 (0.06-0.38)** ^a^	**0.12 (0.03-0.47)**	**<.01** ^a^	**0.39 (0.17-0.91)** ^a^	3.02 (0.67-14.36)	.15	0.67 (0.30-1.48)	0.66 (0.15-2.65)	.57
Marital status									
Currently married	reference	reference	reference	reference	reference	reference	reference	reference	reference
Not currently married	0.89 (0.57-1.40)	0.96 (0.50-1.84)	.90	0.82 (0.44-1.52)	1.27 (0.52-3.21)	.61	0.88 (0.52-1.47)	1.12 (0.54-2.30)	.77
Employment status									
Not currently working	reference	reference	reference	reference	reference	reference	reference	reference	reference
Currently working	0.94 (0.56-1.55)	0.73 (0.35-1.49)	.38	**0.40 (0.16-0.95)** ^a^	2.40 (0.82-8.26)	.13	1.10 (0.62-1.94)	0.62 (0.28-1.33)	.22
Below poverty level									
No	reference	reference	reference	reference	reference	reference	reference	reference	reference
Yes	0.66 (0.41-1.06)	0.88 (0.41-1.89)	.74	**0.43 (0.23-0.79)** ^a^	**0.36 (0.13-0.98)**	**.05** ^a^	0.80 (0.47-1.36)	1.19 (0.53-2.74)	.67
Access to video-enabled device									
No	reference	reference	reference	reference	reference	reference	reference	reference	reference
Yes	0.82 (0.37-1.79)	0.55 (0.15-1.90)	.35	0.85 (0.31-2.33)	0.73 (0.10-4.15)	.74	**0.37 (0.17-0.80)** ^a^	2.35 (0.67-8.24)	.18
Access to internet									
No	reference	reference	reference	reference	reference	reference	reference	reference	reference
Yes	0.97 (0.42-2.25)	1.53 (0.38-6.25)	.55	0.78 (0.23-2.73)	0.84 (0.09-5.15)	.87	**2.79 (1.20-6.49)** ^a^	1.82 (0.43-7.34)	.40
Type of most recent visit									
Video	reference	reference	reference	reference	reference	reference	reference	reference	reference
Telephone	0.67 (0.42-1.06)	**0.52 (0.27-0.99)**	**.05** ^a^	**0.49 (0.24-0.99)** ^a^	0.82 (0.30-2.16)	.69	**0.49 (0.28-0.87)** ^a^	0.59 (0.27-1.24)	.17

Bold indicated *P* < .05.

a Indicates statistically significant differences between groups (*P*-value≤.05).

#### Telemedicine being better or just as good as in-person visits

A total of 145 respondents (42.4%) reported that telehealth was worse than in-person visits or reported being unsure. In univariable analysis, inadequate health literacy (OR 0.60, 95% CI, 0.46-0.98), care at FQHC (OR 0.56, 95% CI, 0.35-0.91), female gender (0.62, 95% CI, 0.39-0.97), Hispanic ethnicity (OR 0.37, 95% CI, 0.20-0.67), and LEP (OR 0.15, 95% CI, 0.06-0.38) were associated with lower likelihood of reporting that telehealth was better or as good as in-person visits. In multivariable models, low patient activation (AOR 0.19, 95% CI, 0.05-0.59) and moderate patient activation (AOR 0.24, 95% CI, 0.07-0.75), female gender (AOR 3.19, 95% CI, 1.67-6.27), LEP (AOR 0.12, 95% CI, 0.03-0.47), and receiving telehealth via telephone (AOR 0.52, 95% CI, 0.27-0.99) were independently associated with lower odds of rating telehealth as better or just as good as in-person visits.

#### Ease of remembering information discussed during telehealth visit

A total of 84.8% participants reported that it was very or somewhat easy to remember information discussed during their telehealth visit. In univariable analyses, participants reporting difficulty remembering information discussed during the telehealth visit were more likely to have inadequate health literacy (OR 0.45, 95% CI, 0.22-0.90), care at an FQHC (OR 0.44, 95% CI, 0.24-0.82), be of Hispanic ethnicity (OR 0.40, 95% CI, 0.20-0.79), have LEP (OR 0.39, 95% CI, 0.17-0.91), not currently working (OR 0.40, 95% CI, 0.23-0.79), and have income below poverty level (OR 0.43, 95% CI, 0.23-0.79). In multivariable models, low patient activation (AOR 0.06, 95% CI, 0.003-0.41) and living below poverty level (AOR 0.36, 95% CI, 0.13-0.98) remained significantly associated with difficulty remembering information during a telehealth visit.

#### Usefulness of telehealth visits once COVID-19 pandemic is over

About 25% of individuals reported that telehealth visits would not be useful postpandemic. In univariable analyses, those who endorsed telehealth to be not useful postpandemic were more likely to have inadequate health literacy (OR 0.47, 95% CI, 0.26-0.84) or reported race as Other (OR 0.26, 95% CI, 0.07-0.90). Technology-related access was also a significant factor, with no access to video-enabled devices (OR 0.37, 95% CI, 0.17-0.80), no access to internet (OR 2.79, 95% CI, 1.20-6.49), and telephone versus video visit (OR 0.49, 95% CI, 0.28-0.87) associated with reporting telehealth visits being not useful once COVID is over. In multivariable models, only having 3 or more chronic conditions remained significantly associated with a higher likelihood to report usefulness of telehealth visits post-COVID (AOR 2.11, 95% CI, 0.99-4.62).

## Discussion

We report on telehealth access and patient experience in a unique diverse cohort with chronic medical conditions and detailed sociodemographic and behavioral data in the Chicago area. Telehealth use was less common among participants of older age, fewer chronic conditions, and living above poverty level. Two-thirds of participants with a telehealth visit had their appointment via telephone versus video. Access to technology, such as video-enabled devices or internet, were barriers to participants having a video visit, as were inadequate health literacy, LEP, and lower SES (eg, less than high school education, income below poverty level).

Participants with a recent telehealth visit typically reported >2 visits within 4 months and overall positive experiences regarding quality (57.6%) and ease of navigating visits (88.9%), similar to results found in a 2020 systematic review which illustrated high patient and provider satisfaction with telehealth during the pandemic.^27^ Furthermore, 80% reported telehealth to be useful during the pandemic, and 75% thought it would be useful postpandemic. Most participants reported it was easy to describe their current health during a telehealth appointment and that it was easy to remember health information discussed during the appointment. Although this highlights the potential benefits and success of continued access to telehealth services, concerns around widening existing health disparities in healthcare access were also highlighted by our data.

Our study highlights continued health disparities across key sociodemographic characteristics (eg, English proficiency, ethnicity, SES, modality of telehealth) in healthcare access and patient experiences of telehealth visits. Prior studies evaluating patient satisfaction of telehealth visits have indicated limited technology access as a contributing factor in patients’ negative perceptions of telehealth quality; however, these studies typically relied on qualitative interviews with small sample sizes, making it difficult to assess for sociodemographic disparities specifically in patient experiences of telehealth.^28–30^ Several studies have evaluated patient disparities in telehealth use and found that older patients, racial and ethnic minorities (eg, Hispanic ethnicity, Black), LEP, and low SES (eg, uninsured, Medicaid insured) were less likely to attend telemedicine appointments.^31–35^ Challenges with technology access, use, and general readiness with telehealth have also been documented, with telehealth “unreadiness” more prevalent in patients who were older, men, not married, Black or Hispanic, had lower SES, and had poorer self-reported health.^36^

In sum, vulnerable populations, such as those with lower health literacy, LEP, low SES, Hispanic ethnicity, receiving care at a FQHC, or telephone visits may be more likely to report having less favorable telehealth experiences (eg, lower quality telehealth visits, less likely to remember telehealth visit information). This may indicate that patients who have greater difficulty understanding health information, whether because of lower health literacy or LEP, may be more likely to report difficulties remembering telehealth information. Thus, telehealth visits also may be less effective for these populations, given the increased likelihood of challenges in remembering telehealth information. Furthermore, perceptions of usefulness of telehealth post-COVID-19, were significantly associated with technology access and modality of telehealth visits (telephone). Patient barriers to technology and internet access, as well as ease of navigating technology, may be important considerations when evaluating reach and effectiveness of telehealth services.

### Limitations

There were several limitations of this study. First, the C3 study surveyed patients who were participants with underlying health conditions actively enrolled in existing, NIH-sponsored studies or clinical trials in one, large US city, which limit the generalizability of findings. Second, this was a cross-sectional analysis of a longitudinal study. Further research is necessary to determine how these health disparities may have potentially changed over the continued pandemic. Third, the telehealth questionnaire did not inquire as to what device or video platform participants used for telehealth. Given that telehealth experiences may likely be influenced by what device and/or video platform a participant uses, this is a limitation of our analysis.

Despite these limitations, this study captured cognitive and psychological determinants of health (eg, CHAI) and their potential impact on patient experiences of telehealth access and use. Currently, limited research studies have been able to capture such detailed psychosocial determinants of health and telehealth PREMs. Recent studies on telehealth adoption and use have either focused on the first few months of the pandemic crisis (January to March 2020), have been specialty- or condition-specific, and/or evaluating synchronous telehealth visits (eg, audio/audiovisual).^3–6^^,^^37–39^ This makes it difficult to make concrete conclusions of effectiveness or on patients’ experiences of telehealth services.

Given the continued use of telehealth services as part of routine healthcare, best practices should target the entirety of the telehealth “ecosystem,” as described in several publications, to better address the structural determinants influencing telehealth care, including provider-specific barriers, accessibility, technology-related barriers, and existing healthcare disparities.^40–44^ Clinicians should consider in-person visits, if feasible, for vulnerable patients who may be more at risk for appointment no-show and/or poorer quality visits when using telehealth (eg, low SES, LEP) or conducting patient visits in another language (ie, Spanish) for patients with LEP. Solutions might also benefit from optimizing patient follow-up, such as sending a summary of the provider’s recommendations immediately after the visit by email, text, or letter; engaging home health or community health workers for follow-up; using home-based diagnostic equipment for follow-up; or connecting the patient with other ancillary professional care (eg, dietician, pharmacist). At a system or institutional-level consideration, also finding opportunities to evaluate a patients’ technology/internet access and internet quality as part of the scheduling process and providing technology onboarding and support as needed.^45^^,^^46^

## Conclusion

COVID has accelerated and revolutionized telehealth, however, disparities in healthcare access and utilization have persisted, and perhaps been exacerbated with telehealth. In particular, patients with lower SES, LEP, and low health literacy may not have high-quality visits and have difficulties retaining instructions and information discussed during their telehealth visit. Health-systems and clinicians need to be aware of these vulnerabilities in order to build effective and equitable telehealth programs.

## Supplementary Material

ooae026_Supplementary_Data

## Data Availability

Individual level data may not be made publicly available due to Institutional Review Board (IRB) and privacy concerns. The data used for this study contain protected health information (PHI) and access is protected by the Northwestern University IRB. The data underlying this article will be shared on reasonable request to the corresponding author.

## References

[1] Crabb DW , Im GY, Szabo G, Mellinger JL, Lucey MR. Diagnosis and treatment of alcohol-associated liver diseases: 2019 Practice Guidance from the American Association for the Study of Liver Diseases. Hepatology. 2020;71(1):306-333. 10.1002/hep.30866 31314133

[2] Gilbert AW , Billany JCT, Adam R, et al Rapid implementation of virtual clinics due to COVID-19: report and early evaluation of a quality improvement initiative. BMJ Open Qual. 2020;9(2):e000985. 10.1136/bmjoq-2020-000985 PMC7247397 32439740

[3] Fisk M , Livingstone A, Pit SW. Telehealth in the context of COVID-19: changing perspectives in Australia, the United Kingdom, and the United States. J Med Internet Res. 2020;22(6):e19264. 10.2196/19264 32463377 PMC7286230

[4] Kaur D , Galloway GK, Oyibo SO. Patient satisfaction with the use of telemedicine in the management of hyperthyroidism. Cureus. 2020;12(8):e9859. 10.7759/cureus.9859 32832308 PMC7437122

[5] Kazi R , Evankovich MR, Liu R, et al Utilization of asynchronous and synchronous teledermatology in a large health care system during the COVID-19 pandemic. Telemed J E Health. 2020;27(7):771-777. 10.1089/tmj.2020.0299 33074786

[6] Morrison KS , Paterson C, Toohey K. The feasibility of exercise interventions delivered via telehealth for people affected by cancer: a rapid review of the literature. Semin Oncol Nurs. 2020;36(6):151092. 10.1016/j.soncn.2020.151092 33223409

[7] Woodall T , Ramage M, LaBruyere JT, McLean W, Tak CR. Telemedicine services during COVID-19: considerations for medically underserved populations. J Rural Health. 2021;37(1):231-234. 10.1111/jrh.12466 32613657 PMC7364549

[8] Veinot TC , Mitchell H, Ancker JS. Good intentions are not enough: how informatics interventions can worsen inequality. *J Am Med Inform Assoc*. 2018;25(8):1080-1088. 10.1093/jamia/ocy052 PMC7646885 29788380

[9] Mobile Fact Sheet: Pew Research Center, 2019. Accessed April 1, 2024. https://www.pewresearch.org/internet/fact-sheet/mobile/

[10] Internet/Broadband Fact Sheet: Pew Research Center, 2019. Accessed April 1, 2024. https://www.pewresearch.org/internet/fact-sheet/internet-broadband/

[11] Koma W , Cubanski J, Neuman T. Medicare and Telehealth: Coverage and Use During the COVID-19 Pandemic and Options for the Future. KFF; 2021.

[12] Anthony DL , Campos-Castillo C, Lim PS. Who isn’t using patient portals and why? Evidence and implications from a national sample of US adults. Health Aff (Millwood). 2018;37(12):1948-1954. 10.1377/hlthaff.2018.05117 30633673

[13] El-Toukhy S , Méndez A, Collins S, Pérez-Stable EJ. Barriers to patient portal access and use: evidence from the health information national trends survey. J Am Board Fam Med. 2020;33(6):953-968. 10.3122/jabfm.2020.06.190402 33219074 PMC7849369

[14] Hughes LD , Done J, Young A. Not 2 old 2 TXT: there is potential to use email and SMS text message healthcare reminders for rheumatology patients up to 65 years old. Health Informatics J. 2011;17(4):266-276. 10.1177/1460458211422019 22193827

[15] Logue MD , Effken JA. An exploratory study of the personal health records adoption model in the older adult with chronic illness. Inform Prim Care. 2012;20(3):151-169. [published Online First: 01/01/2012]. 23710840 10.14236/jhi.v20i3.21

[16] Kruse RL , Koopman RJ, Wakefield BJ, et al Internet use by primary care patients: where is the digital divide? Fam Med. 2012;44(5):342-347. 23027117

[17] Nanda M , Sharma R. A review of patient satisfaction and experience with telemedicine: a virtual solution during and beyond COVID-19 pandemic. Telemed J E Health. 2021;27(12):1325-1331. 10.1089/tmj.2020.0570 33719577

[18] Eze ND , Mateus C, Cravo Oliveira Hashiguchi T. Telemedicine in the OECD: an umbrella review of clinical and cost-effectiveness, patient experience and implementation. PLoS One. 2020;15(8):e0237585. 10.1371/journal.pone.0237585 32790752 PMC7425977

[19] Nguyen M , Waller M, Pandya A, Portnoy J. A review of patient and provider satisfaction with telemedicine. Curr Allergy Asthma Rep. 2020;20(11):72. 10.1007/s11882-020-00969-7 32959158 PMC7505720

[20] Chaudhry H , Nadeem S, Mundi R. How satisfied are patients and surgeons with telemedicine in orthopaedic care during the COVID-19 pandemic? A systematic review and meta-analysis. Clin Orthop Relat Res. 2021;479(1):47-56. 10.1097/corr.0000000000001494 33009231 PMC7899486

[21] Wagner EH , Austin BT, Von Korff M. Organizing care for patients with chronic illness. Milbank Q. 1996;74(4):511-544. 8941260

[22] Barr VJ , Robinson S, Marin-Link B, et al The expanded chronic care model: an integration of concepts and strategies from population health promotion and the chronic care model. Hosp Q. 2003;7(1):73-82. 10.12927/hcq.2003.16763 14674182

[23] Koh HK , Brach C, Harris LM, Parchman ML. A proposed ‘health literate care model’ would constitute a systems approach to improving patients’ engagement in care. Health Aff (Millwood). 2013;32(2):357-367. 10.1377/hlthaff.2012.1205 23381529 PMC5102011

[24] Ratzan S , Parker R. National Library of Medicine Current Bibliographies in Medicine: Health Literacy. National Institutes of Health; 2000.

[25] Kutner M , Greenburg E, Jin Y, Paulsen C. The Health Literacy of America’s Adults: Results from the 2003 National Assessment of Adult Literacy. National Center for Education Statistics; 2006.

[26] Polinski JM , Barker T, Gagliano N, Sussman A, Brennan TA, Shrank WH. Patients’ satisfaction with and preference for telehealth visits. J Gen Intern Med. 2016;31(3):269-275. 10.1007/s11606-015-3489-x 26269131 PMC4762824

[27] Andrews E , Berghofer K, Long J, Prescott A, Caboral-Stevens M. Satisfaction with the use of telehealth during COVID-19: an integrative review. Int J Nurs Stud Adv. 2020;2:100008. 10.1016/j.ijnsa.2020.100008 33083791 PMC7564757

[28] Hendra K , Neemuchwala F, Chan M, Ly NP, Gibb ER. Patient and provider experience with cystic fibrosis telemedicine clinic. Front Pediatr. 2021;9:784692. 10.3389/fped.2021.784692 34900879 PMC8653948

[29] Ladin K , Porteny T, Perugini JM, et al Perceptions of telehealth vs in-Person visits among older adults with advanced kidney disease, care partners, and clinicians. JAMA Netw Open. 2021;4(12):e2137193. 10.1001/jamanetworkopen.2021.37193 34870680 PMC8649833

[30] Nwankwo C , Houpe JE, Ho BVK, Seger EW, Wu DJ, Rajpara A. A multi-site survey study of patient satisfaction with teledermatology. Kans J Med. 2022;15:307-310. 10.17161/kjm.vol15.18073 36196101 PMC9518716

[31] Ryskina KL , Shultz K, Zhou Y, Lautenbach G, Brown RT. Older adults’ access to primary care: gender, racial, and ethnic disparities in telemedicine. J Am Geriatr Soc. 2021;69(10):2732-2740. 10.1111/jgs.17354 34224577 PMC8497419

[32] Schweiberger K , Verma R, Faulds S, Jonassaint CR, White GE, Ray KN. Scheduled and attended pediatric primary care telemedicine appointments during COVID-19. Pediatr Res. 2023;94(1):185-192. 10.1038/s41390-023-02481-w 36690746 PMC9869302

[33] Eberly LA , Kallan MJ, Julien HM, et al Patient characteristics associated with telemedicine access for primary and specialty ambulatory care during the COVID-19 pandemic. JAMA Netw Open. 2020;3(12):e2031640. 10.1001/jamanetworkopen.2020.31640 33372974 PMC7772717

[34] Tong L , George B, Crotty BH, et al Telemedicine and health disparities: association between patient characteristics and telemedicine, in-person, telephone and message-based care during the COVID-19 pandemic. IPEM Transl. 2022;3:100010. 10.1016/j.ipemt.2022.100010 36340828 PMC9617798

[35] Chunara R , Zhao Y, Chen J, et al Telemedicine and healthcare disparities: a cohort study in a large healthcare system in New York city during COVID-19. J Am Med Inform Assoc. 2021;28(1):33-41. 10.1093/jamia/ocaa217 32866264 PMC7499631

[36] Lam K , Lu AD, Shi Y, Covinsky KE. Assessing telemedicine unreadiness among older adults in the United States during the COVID-19 pandemic. JAMA Intern Med. 2020;180(10):1389-1391. 10.1001/jamainternmed.2020.2671 32744593 PMC7400189

[37] Koonin LM , Hoots B, Tsang CA, et al Trends in the use of telehealth during the emergence of the COVID-19 pandemic—United States, January-March 2020. MMWR Morb Mortal Wkly Rep. 2020;69(43):1595-1599. 10.15585/mmwr.mm6943a3 33119561 PMC7641006

[38] Barca I , Novembre D, Giofrè E, et al Telemedicine in oral and maxillo-facial surgery: an effective alternative in post COVID-19 pandemic. Int J Environ Res Public Health. 2020;17(20):7365. 10.3390/ijerph17207365 33050200 PMC7599445

[39] O’Keefe M , White K, Jennings JC. Asynchronous telepsychiatry: a systematic review. J Telemed Telecare. 2019;27(3):137-145. 10.1177/1357633X19867189 31357908

[40] Anaya YB , Bañuelos Mota A, Hernandez GD, Osorio A, Hayes-Bautista DE. Post-pandemic telehealth policy for primary care: an equity perspective. J Am Board Fam Med. 2022;35(3):588-592. 10.3122/jabfm.2022.03.210509 35641044

[41] Saeed SA , Masters RM. Disparities in health care and the digital divide. Curr Psychiatry Rep. 2021;23(9):61. 10.1007/s11920-021-01274-4 34297202 PMC8300069

[42] Simon DA , Shachar C. Telehealth to address health disparities: potential, pitfalls, and paths ahead. J Law Med Ethics. 2021;49(3):415-417. 10.1017/jme.2021.62 34665098

[43] Smith B , Magnani JW. New technologies, new disparities: the intersection of electronic health and digital health literacy. Int J Cardiol. 2019;292:280-282. 10.1016/j.ijcard.2019.05.066 31171391 PMC6660987

[44] SteelFisher GK , McMurtry CL, Caporello H, et al Video telemedicine experiences in COVID-19 were positive, but physicians and patients prefer in-person care for the future. Health Aff (Millwood). 2023;42(4):575-584. 10.1377/hlthaff.2022.01027 37011316 PMC11154740

[45] Choxi H , VanDerSchaaf H, Li Y, Morgan E. Telehealth and the digital divide: identifying potential care gaps in video visit use. J Med Syst. 2022;46(9):58. 10.1007/s10916-022-01843-x 35906432 PMC9361960

[46] Luna P , Lee M, Vergara Greeno R, et al Telehealth care before and during COVID-19: trends and quality in a large health system. JAMIA Open. 2022;5(4):ooac079. 10.1093/jamiaopen/ooac079 36204596 PMC9531686

