# Supplementary material for: Patient factors associated with telehealth quality and experience among adults with chronic conditions
Source: JAMIA Open. 2024 Mar 19;7(2):ooae026. doi: 10.1093/jamiaopen/ooae026 (PMC11000823; doi:10.1093/jamiaopen/ooae026)
Supplement: ooae026_Supplementary_Data [file ooae026_supplementary_data.zip › C3 PREMS_Table 3_2.25.23.docx]

*Table 3. Bivariate and multivariable analyses of factors associated with telehealth PREMs*

|  | **Likelihood of reporting telemed being better or just as good as in-person visits** | | | **Likelihood of reporting very or somewhat easy to remember information discussed during telehealth visit** | | | **Likelihood of reporting usefulness of telehealth visits once COVID is over** | | |
| --- | --- | --- | --- | --- | --- | --- | --- | --- | --- |
| **Participant Characteristics** | **OR (95% CI)** | **AOR (95% CI)** | **p** | **OR (95% CI)** | **AOR (95% CI)** | **p** | **OR (95% CI)** | **AOR (95% CI)** | **p** |
| **Age group** |  |  |  |  |  |  |  |  |  |
| < 60 years |  |  |  |  |  |  |  |  |  |
| 60-69 years | 0.93 (0.55, 1.60) | 9.08 (0.61, 152.34) | 0.50 | 1.03 (0.48, 2.21) | 1.31 (0.47, 3.74) | 0.61 | 0.93 (0.50, 1.71) | 1.19 (0.54, 2.63) | 0.67 |
| > 70 years | 0.90 (0.53, 1.53) | 0.77 (0.36, 1.63) | 0.28 | 0.80 (0.39, 1.65) | 0.62 (0.19, 2.00) | 0.43 | 0.92 (0.50, 1.68) | 2.01 (0.72, 5.90) | 0.19 |
| **CHAI (Patient Activation)** |  |  |  |  |  |  |  |  |  |
| High Activation |  |  |  |  |  |  |  |  |  |
| Moderate Activation | 0.57 (0.23, 1.38) | **0.24 (0.07, 0.75)** | **0.02 **** | 0.30 (0.04, 2.40) | 0.17 (0.01, 1.19) | 0.13 | 1.20 (0.47, 3.09) | 0.83 (0.23, 2.61) | 0.77 |
| Low Activation | 0.45 (0.19, 1.09) | **0.19 (0.05, 0.59)** | **0.01 **** | 0.14 (0.02, 1.07) | **0.06 (0.003, 0.41)** | **0.02 **** | 0.89 (0.35, 2.25) | 0.59 (0.16, 1.86) | 0.39 |
| **Gender** |  |  |  |  |  |  |  |  |  |
| Male |  |  |  |  |  |  |  |  |  |
| Female | **0.62 (0.39, 0.97) **** | **3.19 (1.67, 6.27)** | **< 0.01 **** | 0.62 (0.32, 1.20) | 0.54 (0.19, 1.37) | 0.21 | 0.79 (0.47, 1.34) | 1.09 (0.52, 2.24) | 0.82 |
| **NVS (health literacy)** |  |  |  |  |  |  |  |  |  |
| Adequate (NVS > 4) |  |  |  |  |  |  |  |  |  |
| Inadequate (NVS < 3) | **0.60 (0.36, 0.98) **** | 0.85 (0.41, 1.73) | 0.65 | **0.45 (0.22, 0.90) **** | 0.47 (0.16, 1.32) | 0.16 | **0.47 (0.26, 0.84) **** | 0.48 (0.21, 1.08) | 0.08 |
| **Primary Care Setting** |  |  |  |  |  |  |  |  |  |
| Academic |  |  |  |  |  |  |  |  |  |
| Federally qualified health center | **0.56 (0.35, 0.91) **** | 1.13 (0.47, 2.73) | 0.79 | **0.44 (0.24, 0.82) **** | 0.58 (0.17, 2.01) | 0.39 | 0.64 (0.38, 1.09) | 1.13 (0.44, 2.96) | 0.80 |
| **Health Insurance** |  |  |  |  |  |  |  |  |  |
| Private |  |  |  |  |  |  |  |  |  |
| Medicare or Medicare + Private Supplement | 0.80 (0.41, 1.55) | 0.96 (0.40, 2.27) | 0.93 | 0.54 (0.19, 1.52) | 1.26 (0.32, 4.55) | 0.73 | 0.54 (0.23, 1.28) | 0.62 (0.21, 1.69) | 0.36 |
| Medicaid or Medicaid + Private Supplement | 0.50 (0.25, 1.01) | 0.72 (0.25, 3.75) | 0.52 | 0.42 (0.15, 1.21) | 2.66 (0.59, 11.85) | 0.20 | 0.42 (0.18, 1.01) | 0.64 (0.19, 2.02) | 0.46 |
| **Self-Reported Overall Health** |  |  |  |  |  |  |  |  |  |
| Excellent |  |  |  |  |  |  |  |  |  |
| Very Good | 1.07 (0.43, 2.66) | 1.01 (0.26, 3.75) | 0.99 | 0.99 (0.26, 3.82) | 1.42 (0.15, 8.56) | 0.72 | 0.75 (0.23, 2.44) | 0.83 (0.11, 3.92) | 0.83 |
| Good | 0.96 (0.40, 2.32) | 1.22 (0.33, 4.32) | 0.76 | 1.12 (0.30, 4.16) | 2.04 (0.22, 12.06) | 0.47 | 0.61 (0.19, 1.89) | 0.60 (0.08, 2.63) | 0.54 |
| Fair/Poor | 0.87 (0.35, 2.18) | 1.48 (0.36, 5.89) | 0.58 | 0.41 (0.11, 1.53) | 1.21 (0.13, 7.79) | 0.85 | 0.43 (0.13, 1.39) | 0.56 (0.07, 2.68) | 0.50 |
| **Number of Chronic Conditions** |  |  |  |  |  |  |  |  |  |
| < 3 |  |  |  |  |  |  |  |  |  |
| > 3 | 0.77 (0.49, 1.20) | 0.78 (0.38, 1.54) | 0.48 | 0.64 (0.35, 1.17) | 1.35 (0.51, 3.48) | 0.54 | 0.75 (0.45, 1.23) | **2.11 (0.99, 4.62)** | **0.05**** |
| **Hispanic** |  |  |  |  |  |  |  |  |  |
| No |  |  |  |  |  |  |  |  |  |
| Yes | **0.37 (0.20, 0.67) **** | 0.67 (0.26, 1.71) | 0.39 | **0.40 (0.20, 0.79) **** | 0.36 (0.10, 1.29) | 0.11 | 0.97 (0.49, 1.91) | 1.90 (0.65, 6.54) | 0.27 |
| **Race** |  |  |  |  |  |  |  |  |  |
| Black/African American |  |  |  |  |  |  |  |  |  |
| White/Caucasian | 0.78 (0.46, 1.34) | 1.18 (0.57, 2.45) | 0.65 | 0.60 (0.28, 1.27) | 0.48 (0.15, 1.43) | 0.20 | 1.35 (0.74, 2.49) | 1.23 (0.54, 2.79) | 0.62 |
| Other | 0.43 (0.12, 1.46) | 0.70 (0.15, 3.19) | 0.64 | 1.51 (0.18, 12.94) | 1.14 (0.12, 28.22) | 0.92 | **0.26 (0.07, 0.90) **** | 0.24 (0.05, 1.16) | 0.07 |
| **Highschool Graduate** |  |  |  |  |  |  |  |  |  |
| No |  |  |  |  |  |  |  |  |  |
| Yes | 0.70 (0.42, 1.18) | 1.21 (0.59, 2.46) | 0.60 | 0.55 (0.29, 1.06) | 1.35 (0.53, 3.38) | 0.52 | 0.77 (0.43, 1.38) | 1.09 (0.51, 2.26) | 0.83 |
| **Limited English Proficiency** |  |  |  |  |  |  |  |  |  |
| No |  |  |  |  |  |  |  |  |  |
| Yes | **0.15 (0.06, 0.38) **** | **0.12 (0.03, 0.47)** | **< 0.01 **** | **0.39 (0.17, 0.91) **** | 3.02 (0.67, 14.36) | 0.15 | 0.67 (0.30, 1.48) | 0.66 (0.15, 2.65) | 0.57 |
| **Marital Status** |  |  |  |  |  |  |  |  |  |
| Currently married |  |  |  |  |  |  |  |  |  |
| Not currently married | 0.89 (0.57, 1.40) | 0.96 (0.50, 1.84) | 0.90 | 0.82 (0.44, 1.52) | 1.27 (0.52, 3.21) | 0.61 | 0.88 (0.52, 1.47) | 1.12 (0.54, 2.30) | 0.77 |
| **Employment Status** |  |  |  |  |  |  |  |  |  |
| Not currently working |  |  |  |  |  |  |  |  |  |
| Currently working | 0.94 (0.56, 1.55) | 0.73 (0.35, 1.49) | 0.38 | **0.40 (0.16, 0.95) **** | 2.40 (0.82, 8.26) | 0.13 | 1.10 (0.62, 1.94) | 0.62 (0.28, 1.33) | 0.22 |
| **Below Poverty Level** |  |  |  |  |  |  |  |  |  |
| No |  |  |  |  |  |  |  |  |  |
| Yes | 0.66 (0.41, 1.06) | 0.88 (0.41, 1.89) | 0.74 | **0.43 (0.23, 0.79) **** | **0.36 (0.13, 0.98)** | **0.05 **** | 0.80 (0.47, 1.36) | 1.19 (0.53, 2.74) | 0.67 |
| **Access to video-enabled device** |  |  |  |  |  |  |  |  |  |
| No |  |  |  |  |  |  |  |  |  |
| Yes | 0.82 (0.37, 1.79) | 0.55 (0.15, 1.90) | 0.35 | 0.85 (0.31, 2.33) | 0.73 (0.10, 4.15) | 0.74 | **0.37 (0.17, 0.80) **** | 2.35 (0.67, 8.24) | 0.18 |
| **Access to internet** |  |  |  |  |  |  |  |  |  |
| No |  |  |  |  |  |  |  |  |  |
| Yes | 0.97 (0.42, 2.25) | 1.53 (0.38, 6.25) | 0.55 | 0.78 (0.23, 2.73) | 0.84 (0.09, 5.15) | 0.87 | **2.79 (1.20, 6.49) **** | 1.82 (0.43, 7.34) | 0.40 |
| **Type of most recent visit** |  |  |  |  |  |  |  |  |  |
| Video |  |  |  |  |  |  |  |  |  |
| Telephone | 0.67 (0.42, 1.06) | **0.52 (0.27, 0.99)** | **0.05 **** | **0.49 (0.24, 0.99) **** | 0.82 (0.30, 2.16) | 0.69 | **0.49 (0.28, 0.87) **** | 0.59 (0.27, 1.24) | 0.17 |
| ** indicates statistically significant differences between groups (p-value < 0.05) | | |  |  |  |  |  |  |  |
