# Supplementary material for: Patient factors associated with telehealth quality and experience among adults with chronic conditions
Source: JAMIA Open. 2024 Mar 19;7(2):ooae026. doi: 10.1093/jamiaopen/ooae026 (PMC11000823; doi:10.1093/jamiaopen/ooae026)
Supplement: ooae026_Supplementary_Data [file ooae026_supplementary_data.zip › C3 PREMS_Appendix.docx]

***Appendix Table A. C3 Parent Studies*
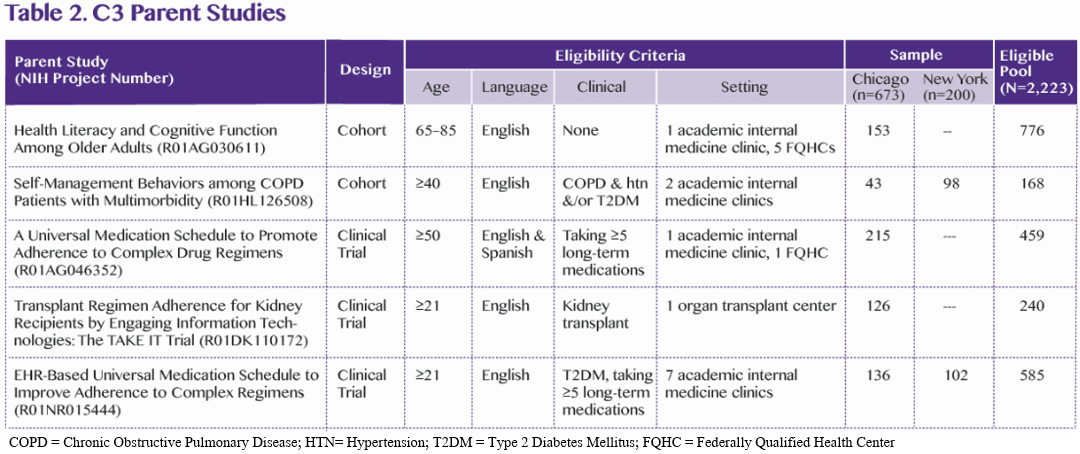
**

***Appendix Table B. C3 Study Measures & Outcomes***


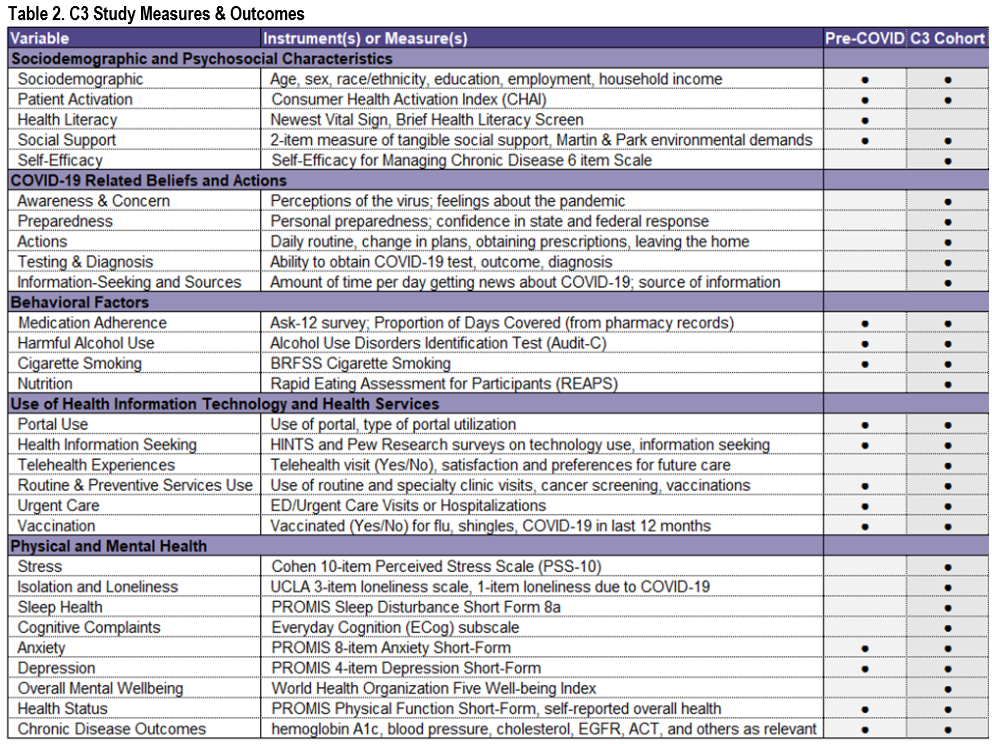


***Appendix Table C. C3 Study Telehealth Patient-Reported Experience Measures***

| **Survey Question** | **Survey Response** | **Collapsed Response (if applicable)** |
| --- | --- | --- |
| Do you, or someone you live with, have a phone, laptop, computer, or tablet that you could use for a video-call with your doctor? | - Yes, Phone - Yes, Laptop/Computer - Yes, Tablet - No | - Yes (phone, laptop/computer, or tablet) - No |
| Are you able to access the internet in your home? This could be through Wi-Fi or a data plan on your phone. | - Yes - No - Don’t know |  |
| In the past 4 months, have you had a telemedicine/telehealth appointment (appointment with your provider by video or phone instead of an in-person appointment)? | - Yes - No - Don’t know |  |
| How many telemedicine visits have you had in the last 4 months? |  |  |
| Was your most recent telemedicine visit done by: | - Telephone - Video |  |
| How did your telemedicine/telehealth visit compare to a traditional in-person medical visit? | - Better than a traditional visit - Just as good - Worse - Not sure | - Better or just as good - Worse or not sure |
| How easy or difficult was it to describe your current health or symptoms during your telehealth visit? | - Very easy - Somewhat easy - Somewhat difficult - Very difficult | - Very or somewhat easy - Very or somewhat difficult |
| How easy or difficult has it been to remember all that was discussed during your telehealth visit? | - Very easy - Somewhat easy - Somewhat difficult - Very difficult | - Very or somewhat easy - Very or somewhat difficult |
| How likely would you be to recommend a telemedicine/telehealth visit to someone else? | - Definitely will - Probably will - Probably will not - Definitely will not | - Would recommend (definitely will or probably will) - Would not recommend (probably will not or definitely will not) |
| How useful have your medical appointments with telemedicine/telehealth been during the COVID pandemic? | - Very useful - Somewhat useful - Neutral - Not very useful | - Useful (very or somewhat useful) - Not useful (neutral or not very useful) |
| How useful do you think it will be to have medical appointments with telemedicine/telehealth after the coronavirus pandemic is over? | - Extremely - Very - Moderately - Slightly - Not at all | - Useful (extremely, very, moderately, or slightly) - Not useful (not at all) |
